# Evaluation of Eight-Item Vancomycin Prescribing Confidence Questionnaire Among Junior Doctors

**DOI:** 10.3389/fmed.2021.677818

**Published:** 2021-05-28

**Authors:** Lu Liu, Zhehan Jiang, Ana Xie, Weimin Wang

**Affiliations:** ^1^Institute of Medical Education, Peking University, Beijing, China; ^2^National Center for Health Professions Education Development, Peking University, Beijing, China

**Keywords:** validity, reliability, antibiotics, psychometrics, assessment

## Abstract

**Background:** Assessing the preparedness of junior doctors to use vancomycin is important in medical education. Preparedness is typically evaluated by self-reported confidence surveys.

**Materials and Methods:** An eight-item vancomycin prescribing confidence questionnaire was developed, piloted, and evaluated. The questionnaire responses were collected from 195 junior doctors and a series of statistical techniques, such as principal component analysis and confirmatory factor analysis, and were implemented to examine the validity and reliability.

**Results:** The principal component analysis supported a one-factor structure, which was fed into a confirmatory factor analysis model resulting in a good fit [comparative fit index (CFI) = 0.99, Tucker–Lewis index (TLI) = 0.99, root mean square error of approximation (RMSEA) = 0.08, standardized root mean square residual (SRMR) = 0.04]. Ordinal-based α was 0.95, and various ωs were all above 0.93, indicating a high reliability level. The questionnaire responses were further proved to be robust to extreme response patterns via item response tree modeling. Jonckheere–Terpstra test results (*z* = 6.5237, *p* = 3.429e−11) showed that vancomycin prescribing confidence differed based on the experience in order (i.e., four ordinal independent groups: “≤10 times,” “11–20 times,” “21–30 times,” and “≥31 times”) and therefore provided external validity evidences for the questionnaire.

**Conclusions:** The questionnaire is valid and reliable such that teaching hospitals can consider using it to assess junior doctors' vancomycin prescribing confidence. Further investigation of the questionnaire can point to the relationship between the prescribing confidence and the actual performance.

## Introduction

Prescribing is more than a physical writing activity because it consists of documenting a history and examination of the patient, determining the cause, planning the therapeutic intervention and objectives of the treatment, communicating with the patient, recording the prescription, and monitoring the consequences (1); it requires a solid understanding of pharmacology, physiology, and clinical evaluation as well as risk appraisal knowledge. Given a fast expansion in both medication availability and multi-morbidity cases, prescribing difficulty becomes increasingly higher nowadays (2).

Being competent in prescribing safely and effectively is essential to a qualified doctor (3, 4); it has been given substantial concerns about how well junior doctors are prepared for prescribing within the context of continuing medical education [CME; (5)]. The UK General Medical Council claimed that nearly 15% of junior doctors felt “not-ready” to handle clinical problems far above their skill and knowledge level, while 10% of the group believed that they were not ready for choosing medicines like antibiotics (6). What is more, that medical students are not competent in prescribing antibiotics when the basic medical training finishes is a global concern (7–9). Among these medicines, prescribing vancomycin is regarded challenging in practice, because it requires dosing levels and serum drug levels to vary from case to case, in the perspectives of efficacy and toxicity (10, 11). To evaluate CME antimicrobial stewardship, teaching hospitals are likely to assess the junior doctors' confidence in prescribing and monitoring antibiotics. As “confidence” is a latent trait, survey approaches are generally adopted to collect data for that invisible inquiry.

Assessment surveys based upon a Likert-type rating scale, among all instruments, are used dominantly in the broad field of medical education to answer questions that can be difficult/impossible to obtain via direct observations (12). Phillips et al. (13) created a vancomycin prescribing confidence questionnaire that consisted of eight items, of which the content validity was assessed by six pharmacists and physicians.

However, a systematic evaluation of the questionnaire, such as reliability and validity studies, had not achieved yet. Further, in a questionnaire of this kind, studies have found that respondents may show inclinations to favor or avoid certain response categories, which contaminate the latent traits of interest and produce biased results consequently (14); for example, affected by some emotional stimulus, a respondent whose true latent trait is “Disagree” keeps selecting “Strongly Disagree” across survey items. Therefore, this article validates the use of the questionnaire via a series of statistical analyses, such that teaching hospitals can implement it for the prescribing confidence assessment.

## Methods

**Figure 1:**
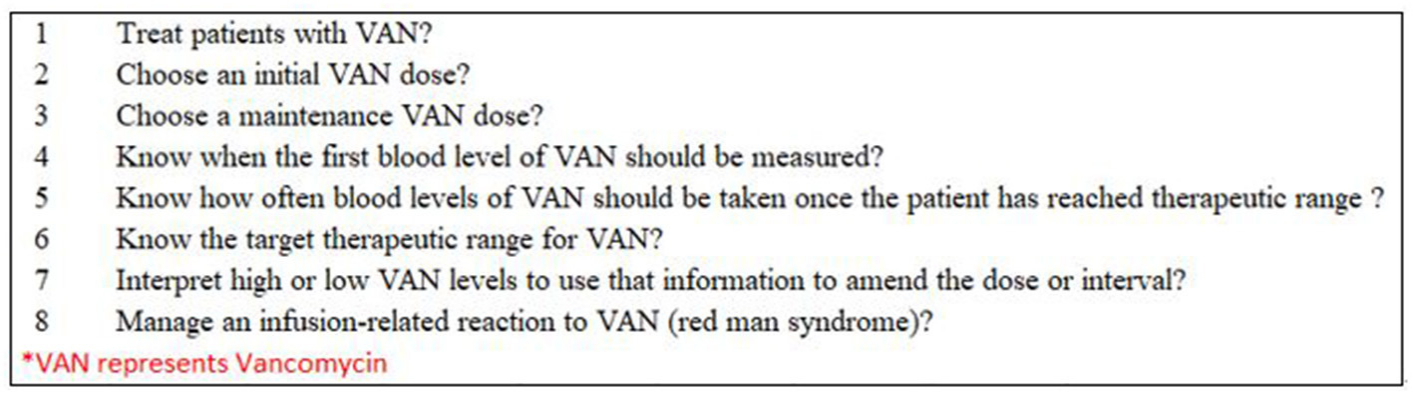
The eight items of the assessment survey for vancomycin prescribing confidence.

The questionnaire was distributed to 195 junior doctors working at Flinders Medical Center in Australia to assess their confidence about prescribing and monitoring vancomycin, where the questions are listed in Figure 1 and the response categories are in a 5-point Likert scale of “Strongly Disagree,” “Disagree,” “Neutral,” “Agree,” and “Strongly Agree” [see (13), for the dataset]. All of present analyses treated the responses as ordinal values instead of numeric ones; this practice has been proved to be more methodologically appropriate than those conventionally assuming Likert-scale responses are continuous (15). Further, the respondents' practice experience in antibiotics prescribing was also collected: “≤10 times,” “11–20 times,” “21–30 times,” or “≥31 times.”

The R software was used to perform all the analyses, and the R script is attached to Appendix I. Out of the dataset's 1,755 data points, 10 missing values were found and imputed via hot deck method such that missing cells were replaced with an observed data point from a “similar” unit (16). A principal component analysis (PCA) was performed to investigate the potential number of dominant factors. An ordinal confirmatory factor analysis [CFA; (17)] was fitted to provide both factorial and construct evidence: the parameter estimates and the model fit were examined. Further, the latent traits were extracted and ranked by the prescribing experience; Jonckheere–Terpstra monotonic trend test was deployed to investigate the possible existence of a trait-level ascending trend as the experience increases (18). To test if the questionnaire was robust to extreme response styles, an item response tree model—a modeling paradigm for investigating the undesirable response patterns—was fitted and compared with those yielded by a regular item response theory model (19–21). In addition, ordinal-based α was implemented to demonstrate the reliability level rather than Cronbach's α, given the response characteristics (22). The reliability evidence was also collected from three omegas: ω_1_ (23), ω_2_ (24), and ω_3_ (25).

## Ethics

*Ethics committee approval*: Ethical review and approval were not required for the study on human participants in accordance with the local legislation and institutional requirements. *Consent procedures*: Written informed consent from the junior doctors working at Flinders Medical Center was not required to participate in this study in accordance with the national legislation and the institutional requirements.

## Results

The responses were collected during 2012 to 2014 via two waves, of which the first recruited 120 participants and the second one involved 75. Grouped by their postgraduate years (e.g., PGY1 and PGY2 represent the first and second postgraduate years), there were 188 PGY1 as well as seven PGY2 and above junior doctors. In terms of their experience in antibiotics prescribing, 157 participants selected “≤10 times,” 25 participants chose “11–20 times,” eight participants checked “21–30 times,” and three participants endorsed “≥31 times.”

**Figure 2:**
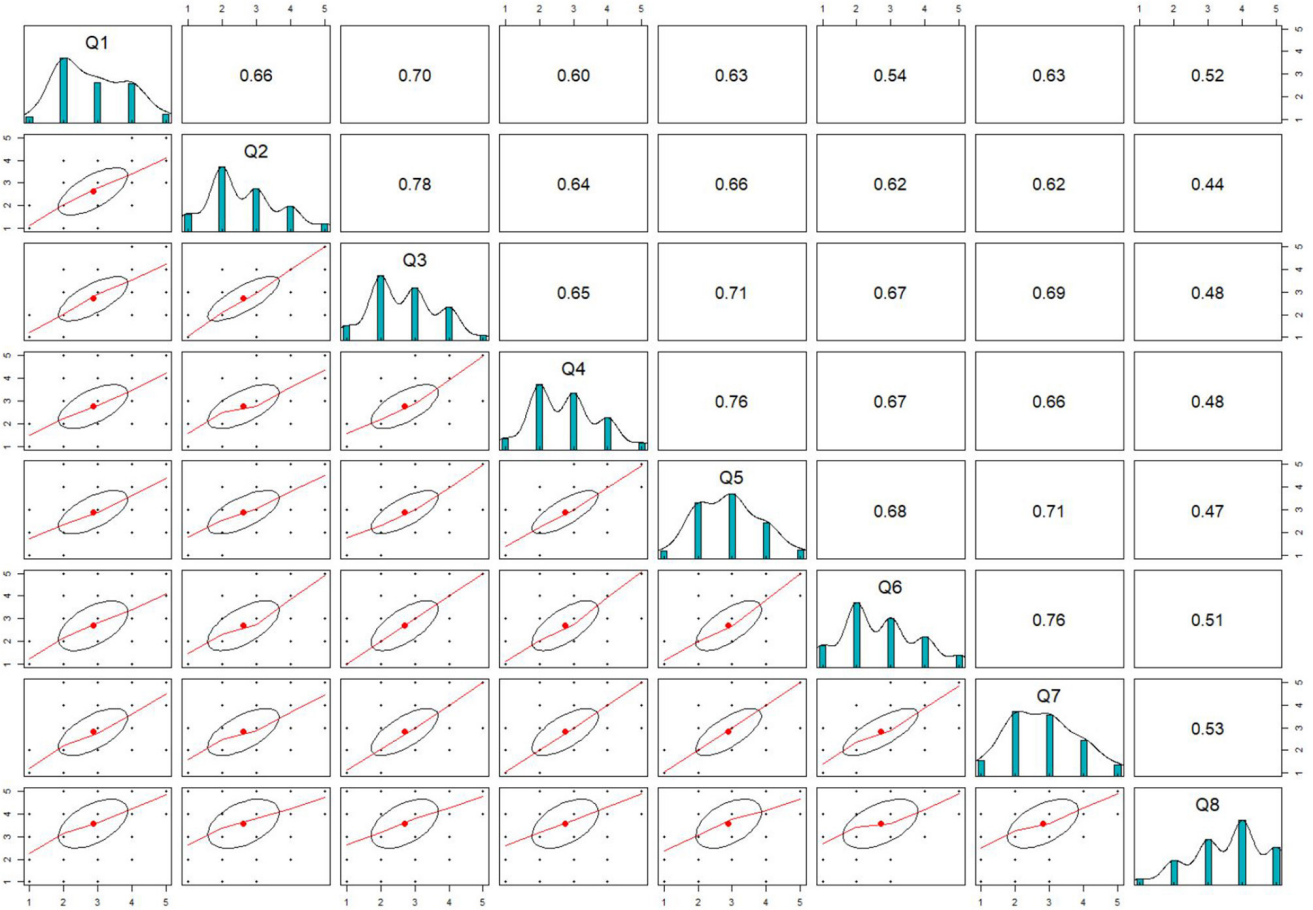
The visualization of the descriptive statistics for the eight items.

Figure 2 shows distributions of responses for each individual question as well as the pairwise correlations. If two variables are correlated as high as 0.9 or above, they are regarded as redundant statistically; on the other hand, if the correlation is too low (e.g., 0.2), they are not essentially good indicator-pairs serving for the same measure. It can be seen that the questions were well-correlated to an appropriate degree, as the span of the correlations ranged from 0.44 to 0.78. All items except Q5 and Q8 were right-skewed; these corresponded to their mean values (2.872, 2.629, 2.703, 2.754, 2.896, 2.694, 2.808, 3.572) where Q8 ranked the highest. In terms of variance, the result of (1.030 1.090, 0.963, 0.939, 0.907, 1.210, 1.075, 1.175) indicated that Q8 reflected the largest variability among all.

After hot deck imputation, the PCA yielded a one-factor structure as (1) the proportion of variance explained by one component was 0.70, (2) the correlation of (regression) scores with factors was 0.98, (3) multiple *R*^2^ of scores with factors was 0.95, and (4) minimum correlation of possible factor scores was 0.91. The results meant that one factor is adequate to contain the main body of the entire dataset, meaning that the questionnaire is indeed appropriate to measure “prescribing confidence” as a latent variable of interest. The conclusion about the one-factor structure was then forwarded to the CFA model.

The model fit values indicated that the CFA model was appropriate (26, 27): χ^2^ = 11, 683.474 (df = 28 and *p*-value < 0.000), comparative fit index (CFI) = 0.99, Tucker–Lewis index (TLI) = 0.99, standardized root mean square residual (SRMR) = 0.04, and root mean square error of approximation (RMSEA) = 0.08. The good fit results provided evidences supporting that the questionnaire had acceptable construct validity. As seen in Table 1, all the standardized loadings were >0.6 and statistically significant, as their *p*-values were all below 0.05; the loading estimates supported a good factorial validity level of the questionnaire (28). Q8 is loading the smallest among all; this finding corresponds to the fact that it correlated less with other variables as seen in Figure 2. Overall, the factor loadings show that the items were capable of discriminating the latent variable and therefore are good indicators for the measure.

**Table 1:** Loading estimates of the confirmatory factor analysis modeling.

**Item**	**Estimate**	**Std.Err**	**z-value**	***p*(**>|**z**|**)**
Q1	0.821	0.028	29.461	0.000
Q2	0.867	0.018	46.917	0.000
Q3	0.912	0.015	62.427	0.000
Q4	0.862	0.021	40.269	0.000
Q5	0.884	0.018	48.074	0.000
Q6	0.841	0.021	39.836	0.000
Q7	0.882	0.020	44.026	0.000
Q8	0.632	0.045	14.147	0.000

The averages of the latent traits extracted from the CFA model for “≤10 times,” “11–20 times,” “21–30 times,” and “≥31 times” groups were −1.624, −1.056, −0.575, and 0.175, respectively; Jonckheere–Terpstra test yielded a significant result with *z* = 6.5237 and *p*-value = 3.429e−11, implying a monotonically ascending trend of vancomycin prescribing confidence when more experience is expected theoretically; the result served as evidence for an appropriate level of external validity.

The indices of Akaike information criterion (AIC), Bayesian information criterion (BIC), and corrected AIC (AICC) for the item response tree model and the regular item response model (i.e., graded response model in the analysis) were (3,391, 3,489, 3,402) and (3,346, 3,477, 3,367), respectively; as lower indices imply a better model fit, the regular item response model was preferred over the tree one, implying that no considerable extreme response styles were detected from the questionnaire. This conclusion was further verified by the correlation between two models' latent trait estimates: the correlation was 0.873 and its 95% confidence interval was (0.835, 0.903). This part indicated that the questionnaire had acceptable response process validity.

Last but not least, the questionnaire was proved to be reliable as several reliability indexes turned out to be high. Ordinal-based α was 0.95, which was above the conventional threshold −0.9. Similarly, the ω_1_, ω_2_, and ω_3_ estimates were 0.933, 0.933, and 0.946, respectively.

## Discussion

This is the first attempt to systemically evaluate the validity and reliability of the eight-item questionnaire designed for assessing junior doctors' preparedness to prescribe the antibiotic vancomycin. Specifically, the study was achieved via various statistical techniques chained through a psychometric perspective. The PCA results indicated that the questionnaire was one-factor structured; the CFA results showed good factorial and construct validity; Jonckheere–Terpstra test results demonstrate appropriate external validity matching theoretical expectation; the item response tree modeling results proved that the questionnaire was robust to extreme response patterns; many reliability indexes led to a conclusion that the questionnaire was reliable and consistent.

Antibiotic misuse, along with a reduction in the number of new medications entering the pharmacy, has resulted in the growth of antibiotic-resistant bacteria. To avoid this, antibiotics should only be used as absolutely necessary, and the whole drug should be ingested. Therefore, doctors are expected to master appropriate levels of the knowledge to ensure patients' health-care quality. The questionnaire provides a solution to reflecting junior doctors' confidence in the prescribing confidence; it may be considered a threshold of the readiness for independently prescribing.

The limits of this study are evident: the samples were collected from one hospital only, and the external validity remains unstudied. Further studies should triangulate the questionnaire with actual performance indicators to show how the confidence varies with performance, which can be in the forms of multiple-choice test and clinical skill examinations. In addition, the questionnaire can be extended to general prescribing practice, while corresponding validations are needed prior to the further applications.

## Conclusions

With all the evidence pointing to the fact that the questionnaire can function validly and reliably, teaching hospitals can consider adopting the questionnaire in the future practice to evaluate CME antimicrobial stewardship, specifically assessing the junior doctors' confidence in prescribing and monitoring antibiotics in the present context.

## Data Availability Statement

The datasets presented in this study can be found in online repositories. The names of the repository/repositories and accession number(s) can be found below: https://doi.org/10.7910/DVN/GLCXW5.

## Ethics Statement

Ethical approval was not provided for this study on human participants because the study is based upon secondary data analysis. The patients/participants provided their written informed consent to participate in this study.

## Author Contributions

ZJ, LL, and AX proposed the idea together, while ZJ and WW handled both the design and the data analysis. LL as well as AX conducted the literature review and the discussion. All authors contributed to the article and approved the submitted version.

## Conflict of Interest

The authors declare that the research was conducted in the absence of any commercial or financial relationships that could be construed as a potential conflict of interest.
