# Supplementary material for: Evaluation of Eight-Item Vancomycin Prescribing Confidence Questionnaire Among Junior Doctors
Source: Front Med (Lausanne). 2021 May 28;8:677818. doi: 10.3389/fmed.2021.677818 (PMC8193050; doi:10.3389/fmed.2021.677818)
Supplement: Supplementary file 1 [file Data_Sheet_1.docx]

**Appendix I**

**library(tidyr)**

**library(hot.deck)**

**library("ItemResponseTrees")**

**library(psych)**

**library(ggplot2)**

**ggplot(data = mpg, mapping = aes(x = condition, y = measurement)) +**

**geom_boxplot()**

**library(psych)**

**summary(dat[,1:8])**

**pairs.panels(dat[,1:8],**

**method = "pearson", # correlation method**

**hist.col = "#00AFBB",**

**density = TRUE, # show density plots**

**ellipses = TRUE # show correlation ellipses**

**)**

**ggplot(data = mpg) +**

**geom_boxplot(mapping = aes(x = reorder(mpg,condition, measurement, FUN = median), y = measurement)) +**

**coord_flip()**

**V <- length(vars)**

**dat<-hot.deck(dat)**

**dat<-dat[[1]]**

**dat<-dat[[1]]**

**summary(dat)**

**pca.res<-(fa.poly(dat[,1:8],))**

**fa.diagram(pca.res)**

**fa.parallel(dat[,1:8], fm="pa", fa="fa", main = "Scree Plot")**

**dat_long<-gather(dat, Item, Response, Q1:Q8)**

**dat_long$Response<-as.factor(dat_long$Response)**

**dat_long$experience<-as.factor(dat_long$experience)**

**dat_long$Group<-as.factor(dat_long$Group)**

**#IRTree Model**

**m1 <- "**

**# IR-tree model for 5-point items (B鯿kenholt, 2012)**

**Equations:**

**1 = (1-m)*(1-t)*e**

**2 = (1-m)*(1-t)*(1-e)**

**3 = m**

**4 = (1-m)*t*(1-e)**

**5 = (1-m)*t*e**

**IRT:**

**t BY Q1@1, Q2@1, Q3@1, Q4@1, Q5@1, Q6@1, Q7@1, Q8@1;**

**e BY Q1@1, Q2@1, Q3@1, Q4@1, Q5@1, Q6@1, Q7@1, Q8@1;**

**m BY Q1@1, Q2@1, Q3@1, Q4@1, Q5@1, Q6@1, Q7@1, Q8@1;**

**Class:**

**Tree**

**"**

**model1 <- irtree_model(m1)**

**ctrl <- control_mirt(method = "MHRM",control = list(iterations=400000))**

**fit1 <- fit(model1, data = dat[,1:8], engine = "mirt")**

**tidy(fit1, par_type = "difficulty")**

**glance(fit1)**

**augment(fit1)**

**library("mirt")**

**#GRM Specificiation**

**m2 <- "**

**# Graded response model**

**IRT:**

**t BY Q1, Q2, Q3, Q4, Q5, Q6, Q7, Q8;**

**Class:**

**GRM**

**"**

**model.grm <- 'liking.science = 1-8'**

**results.grm <- mirt(data=dat[,1:8], model=2, itemtype="graded", SE=TRUE, verbose=FALSE,calcNull = TRUE)**

**coef.grm<-coef(results.grm,IRTpars=TRUE, simplify=TRUE)**

**items.grm <- as.data.frame(coef.grm$items)**

**print(items.grm)**

**M2(results.grm)**

**summary(dat)**

**model2 <- irtree_model(m2)**

**library(lavaan)**

**HS.model <- ' confidence =~ NA*Q1 + Q2 + Q3 + Q4 + Q5 + Q6 + Q7 + Q8**

**confidence ~~ 1*confidence '**

**fit <- cfa(HS.model, data=dat[,1:8],ordered=c("Q1","Q2","Q3","Q4","Q5","Q6","Q7","Q8"))**

**#fit <- cfa(HS.model, data=dat[,1:8])**

**summary(fit, fit.measures=TRUE)**

**fitMeasures(fit)**

**AIC(fit)**

**#Compare**

**rbind(glance(fit1), glance(fit2))**

**model1$mapping_matrix**

**model2$mapping_matrix**

**#IRTree Model Results**

**options(tibble.print_max = Inf)**

**tidy(fit1, par_type = "difficulty")**

**glance(fit1)**

**augment(fit1)[,9:14]**

**fit2 <- fit(model2, data = dat[,1:8], engine = "tam")**

**rbind(glance(fit1), glance(fit2))**

**glance(fit2)**

**augment(fit2)[,9]**

**mean(unlist(augment(fit2)[,9])[dat$experience==1])**

**mean(unlist(augment(fit2)[,9])[dat$experience==2])**

**mean(unlist(augment(fit2)[,9])[dat$experience==3])**

**mean(unlist(augment(fit2)[,9])[dat$experience==4])**

**#Test**

**(jonckheere.test(unlist(augment(fit2)[,9]) , as.factor(dat$experience), "decreasing"))**

**(jonckheere.test(unlist(predict(fit)) , as.factor(dat$experience), "decreasing"))**

**#Alpha**

**ABC.pc <- polychoric(dat[,1:8])**

**alpha(ABC.pc$rho)**

**library(semTools)**

**reliability(fit, return.total = FALSE, dropSingle = TRUE,**

**omit.imps = c("no.conv", "no.se"))**
